# Soil Microbial Blueprint: Predicting Soil Dominant Bacterial Genera Distribution Across Australia

**DOI:** 10.1111/mec.70135

**Published:** 2025-10-04

**Authors:** Mingming Du, Peipei Xue, Budiman Minasny, Alex McBratney, Mario Fajardo Pedraza, Vanessa Pino, Patrice de Caritat, JiZheng He, Qinglin Chen, Andrew Bissett

**Affiliations:** ^1^ School of Life and Environmental Sciences The University of Sydney Camperdown New South Wales Australia; ^2^ Sydney Institute of Agriculture The University of Sydney Eveleigh New South Wales Australia; ^3^ John de Laeter Centre Curtin University Bentley Western Australia Australia; ^4^ School of Agriculture, Food and Ecosystem Sciences, Faculty of Science The University of Melbourne Parkville Victoria Australia; ^5^ CSIRO Hobart Tasmania Australia

**Keywords:** climate, digital mapping, dominant bacterial genera, land use

## Abstract

Soil bacteria play a crucial role in soil processes, such as carbon sequestration and nutrient cycling. While soil bacterial communities and their interactions with pedo‐climatic factors have been well documented, most studies typically focus on broad taxonomic levels, leaving distribution and responses at the genus level unexplored. This study optimized machine learning models to predict the distribution of dominant bacterial genera across Australia on a comprehensive dataset of 1971 topsoil samples. Our high‐resolution digital maps (~1 km resolution) reveal four distinct distribution patterns for the dominant bacterial genera: coastal or inland enriched patterns and latitude‐related patterns. Each genus exhibited unique responses to critical factors, including temperature, precipitation, soil organic carbon (SOC), and pH. Notably, our findings highlight the importance of genus‐level analysis, as bacterial genera within the same phylum can respond markedly differently to pedo‐climatic conditions. Intensive land use significantly homogenized bacteria composition and increased the relative abundance of Rubrobacter, RB41, Microvirga, and Sphingomonas. Overall, this study enhances our understanding of bacterial macroecological trends and offers insights for more precise interventions to improve soil health and resilience against environmental changes.

## 1 Introduction

Soil bacteria are among the most abundant microorganisms in soil ecosystems (Banerjee and Van Der Heijden 2023) and constitute a significant portion of terrestrial biodiversity. They play crucial roles in nutrient cycling, carbon sequestration, and overall ecosystem productivity (Delgado‐Baquerizo et al. 2016). Their rapid reproduction and ability to adapt to diverse environmental conditions, due to high mutation rates and genetic recombination (Roszak and Colwell 1987), complicate our understanding of their ecological preferences and biogeographic patterns.

Specific bacterial taxa thrive in distinct ecological niches shaped by various environmental factors (Delgado‐Baquerizo et al. 2017). Extensive research over the past few decades has mapped the biogeographic patterns of soil bacteria at regional (Wang et al. 2017), continental (Xue et al. 2024), and global scales (Delgado‐Baquerizo et al. 2018). These studies have demonstrated that the spatial distribution of topsoil microbes is driven by both biotic and abiotic factors, including climatic conditions (Delgado‐Baquerizo et al. 2017; Du et al. 2025), soil properties (Philippot et al. 2024), geographic distance (Li et al. 2020), vegetation cover (Li et al. 2020), and topographic factors (Peng et al. 2019). A pioneering study in the United States revealed that soil characteristics, particularly pH, are the most influential factors affecting bacterial diversity (Fierer and Jackson 2006), consistently supported by subsequent research (Berdugo et al. 2020; Mod et al. 2021). However, the response of local soil conditions to broader environmental influences remains uncertain and may lead to shifts in soil pH (Mod et al. 2021). For instance, a global survey demonstrated that aridity can have a profound impact on soil pH, indirectly affecting both bacterial diversity and abundance (Fierer and Jackson 2006). While these studies offer valuable insights into the environmental drivers of soil microbial community structure, most have focused on bacterial richness and diversity (Li et al. 2020; Peng et al. 2019).

Thus, significant gaps remain in understanding the biogeography of specific bacterial taxa and the mechanisms underlying their distribution. While previous studies have explored global soil bacterial distribution patterns (Crowther et al. 2019; Delgado‐Baquerizo et al. 2018), they often centred on broad ecological clusters, without examining finer‐scale patterns. Our earlier work successfully predicted a bacterial phylum distribution map across Australia (Xue et al. 2024); however, such broad taxonomic resolution may overlook ecological nuances. For example, within the Acidobacteriota phylum, the same environmental drivers can elicit divergent responses among subgroups: higher pH levels promote subgroups 1, 2 and 3 but reduce the abundance of subgroups 4 and 6 (Jones et al. 2009; Rousk et al. 2010). These complexities highlight the need for finer taxonomic resolution, as emphasised by recent advances in microbial research (Walsh et al. 2019). Furthermore, a study in France predicted the dominant genera within a specific phylum but showed limited model accuracy (*r* ^2^ < 0.3) (Karimi et al. 2018). Achieving a more accurate understanding of bacterial genus distribution is essential for identifying their ecological preferences and assessing their vulnerabilities to global environmental change.

Recent advancements in statistical modelling and machine learning have enabled soil scientists to link soil and climatic factors with microbial spatial distributions (Mod et al. 2021; Pino et al. 2023; Xue et al. 2024). However, previous studies often relied on limited soil samples to predict bacterial distribution at national or global scales (Fierer and Jackson 2006; Serna‐Chavez et al. 2013), restricting their ability to capture fine‐scale environmental influences. This study addresses the gaps in soil bacterial genus research by analysing topsoil samples from 1971 locations across Australia. We evaluate the continental‐scale influences of soil properties, vegetation cover, climate, and geographic distance on the distribution of soil bacterial genera. We hypothesise that dominant bacterial genera exhibit distinct distribution patterns across Australia. We employ machine learning algorithms to identify the key factors shaping bacterial genus distribution and develop high‐resolution digital maps. This continental spatial analysis enhances our understanding of bacterial genus environmental preferences and provides insights for designing future surveys of soil biodiversity and ecosystem function.

## 2 Materials and Methods

### 2.1 Study Area

We compiled a comprehensive dataset of soil bacteria by integrating multiple publicly available sources and our own prior research. These included: the Biomes of Australian Soil Environments (BASE) (Bissett et al. 2016) with PRJNA316147 sequenced with primers 27F–519R (V1–V3); the National Geochemical Survey of Australia (NGSA) (Caritat 2022) and Northern Australia Geochemical Survey (NAGS) (Bastrakov and Main 2020), with PRJNA690062 sequenced with 27F–519R (V1–V3); a dataset from the National Centre for Biotechnology Information (NCBI) (Yan et al. 2021), with PRJNA659980 sequenced with 515F‐modF/806R‐modR (V4); data from our previous studies (Pino et al. 2019) with PRJNA729592 sequenced with 341F/805R (V3–V4) and our recent investigation across New South Wales (NSW) (Du et al. 2025) with PRJNA1281203 sequenced with 341F/805R (V3–V4).

**FIGURE 1:**
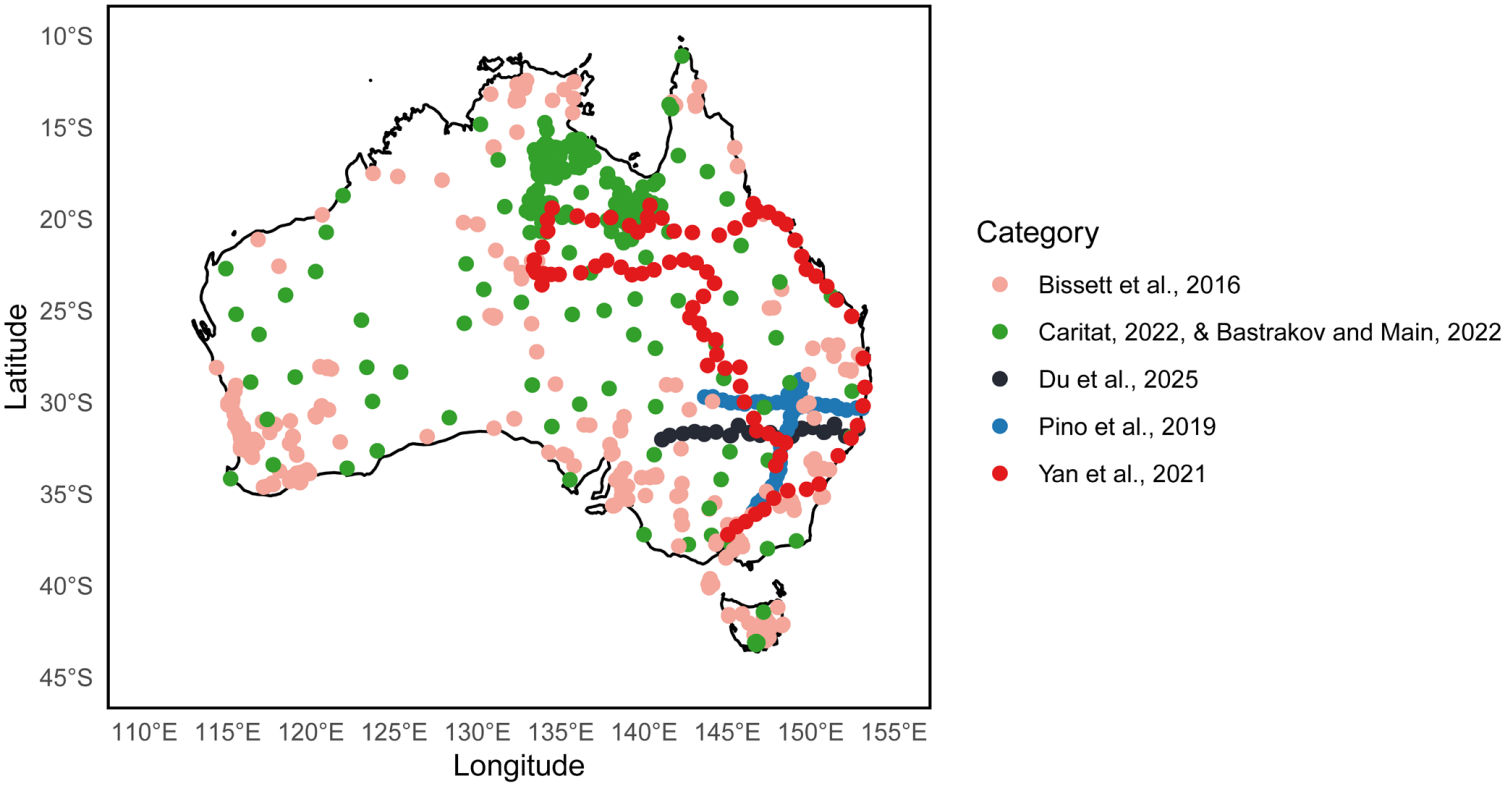
Locations of the 1971 sampling sites across Australia coloured by database.

For the analysis focusing on the distribution of topsoil bacteria, we selected soil samples taken from depths of 0–15 cm. In total, we compiled 1971 samples: 1095 from the BASE, 289 from NGSA and NAGS, 192 from NCBI, and 395 from our prior research (Figure 1). To our knowledge, this study represents the most extensive coverage of soil bacterial data across Australia.

### 2.2 Soil Physicochemical Characterisation and Climatic Data Acquisition

To explain the spatial pattern of the bacteria, we considered key soil physicochemical factors, including clay content, sand content, pH, soil organic carbon (SOC), cation exchange capacity (CEC), and phosphorus (P) concentrations. Climatic factors, including annual daily mean temperature (MAT), mean annual precipitation (MAP), and annual temperature range (TRA), were also considered. Vegetation was assessed using the Normalised Difference Vegetation Index (NDVI) for each annual quarter (Q1, Q2, Q3 and Q4), and topographic variables include elevation and slope. These factors represent long‐term averages of the soil, climate, and vegetation conditions.

These soil and environmental factors were derived from 15 covariate raster layers with a resolution of 90 m containing pedo‐climatic information and land use information from the Terrestrial Ecosystems Research Network (TERN) via the ‘SLGACloud’ package in R (Xue et al. 2024). TERN measures key terrestrial ecosystems based on remote sensing techniques, providing environmental data over time. We extracted all factors based on geographic coordinates using the ‘raster’ package in R.

Briefly, our 1971 samples encompass a diverse range of Australian pedo‐climatic variables: soil pH ranged from 4.0 to 9.0, SOC content from 0.4% to 13.6%, MAP from 155 to 3440 mm, and MAT from 12.2°C to 34.4°C. For detailed information on the land use classification, please refer to Table S1. Briefly, our four land use categories represent a gradient of human impact: Natural land covers minimally disturbed ecosystems; Relative Natural includes areas with low‐intensity human interaction; Dryland encompasses rain‐fed agricultural practices often found in arid and semi‐arid regions; and Intensive Use represents areas with high‐intensity human modification.

### 2.3 Molecular Biology

To characterise the soil bacterial community composition, we utilised 16S rRNA gene sequencing data. Briefly, raw sequencing data were first quality‐checked using FastQC and then merged with FLASH (Magoč and Salzberg 2011). Operational Taxonomic Units (OTUs) were clustered at a 97% sequence similarity threshold using USEARCH (v8.0.1517). Taxonomic classification of OTUs was performed using the SILVA 138 database (Quast et al. 2012). Though combining the different datasets may introduce bias, the use of multi‐primer datasets for bacterial biogeographic patterns is still a valid method (Varliero et al. 2023). To minimise bias from extremely rare taxa, we filtered out bacterial OTUs with fewer than 10 total reads or those observed in fewer than two samples. We then rarefied the OTU table to ensure even sequencing depth across samples. To accommodate the substantial variation in sequencing depth across our four datasets, we applied dataset‐specific rarefaction depths as follows: PRJNA316147 and PRJNA690062 were rarefied to 10,083 reads per sample, PRJNA659980 to 25,506 reads, PRJNA729592 to 1000 reads, and PRJNA1281203 to 9234 reads.

Dominant genera were identified based on the mean relative abundance greater than 0.08% and occurrence in more than 50% of samples. This criterion was based on previous studies, with an adjustment tailored to our data (Xue et al. 2024). Based on this criterion, we obtained 16 dominant genera, accounting for 25.9% of the total relative abundance.

### 2.4 Data Analysis

We plotted genus‐level frequency of occurrence against mean relative abundance (log_10_‐transformed) and fitted a nonparametric loess smooth and calculated *R* ^2^ as one minus the ratio of the residual sum of squares. To examine the relationship between whole bacterial genera and environmental drivers, we conducted Canonical Correspondence Analysis (CCA) using the ‘vegan’ package in R (Oksanen et al. 2013). We coloured the points according to the MAP based on the squared correlation of the environmental variables.

To identify non‐linear response patterns of dominant bacterial genera to pedo‐climatic factors, we used the ‘treeshap’ package (Yang 2021) to compute Shapley Additive Explanations (SHAP) values, elucidating the contribution of each variable. We assessed the effects of land use on dominant bacterial genera and phylum‐level communities through principal coordinates analysis (PCoA) biplots based on Bray–Curtis distances, also using the 'vegan' package. Additionally, we created heatmaps using the ‘pheatmap’ package (Kolde and Kolde 2015) to visualise the impact of land use on the relative abundance of dominant bacterial genera. We also generated boxplots and performed one‐way ANOVA to investigate differences in pedo‐climatic factors among various land use types.

### 2.5 Modelling

We used the digital soil mapping framework to link microbial observations to environmental factors via machine learning (Ma et al. 2019; Minasny et al. 2024). We employed a random forest (RF) model to assess the relationship between environmental factors and dominant bacterial taxa (Bahram et al. 2018). The covariates included soil properties (SOC, pH, sand, clay, CEC, P), climatic variables (MAP, MAT, TRA), vegetation coverage (NDVI_Q1, NDVI_Q2, NDVI_Q3, NDVI_Q4), and topographic features (slope, elevation). To mitigate multicollinearity, we calculated variance inflation factors (VIFs) for all 15 covariates and applied a conservative threshold of VIF ≤ 10 (Kasraei et al. 2024). Covariates exceeding this threshold were excluded, leaving a final set of 10 uncorrelated predictors: P, pH, SOC, CEC, MAP, MAT, TRA, elevation, slope and NDVI_Q3. The relative abundance of each dominant bacterial genus served as the response variable in the model.

Model evaluation was conducted using a 10‐fold cross‐validation approach (Xue et al. 2024). The dataset was randomly divided into 10 equal subsets. For each iteration, nine subsets were used for training, and the remaining subset was used for validation. This process was repeated 10 times, ensuring each subset was used for validation once. We calculated validation statistics, including the Root Mean Squared Error (RMSE), coefficient of determination (*R* ^2^), and Concordance Correlation Coefficient (*ρ*_C_), across the entire dataset to assess model performance.

Based on model performance, we selected the most influential environmental covariates for predicting the distribution of dominant bacterial genera (McBratney et al. 2003). We then used the Quantile Regression Forest (QRF) model for spatial prediction (Mahjenabadi et al. 2022). The QRF model parameters were set as follows: number of trees = 250, minimum node size = 5, and mtry (number of variables tried at each split) equal to the square root of the total covariates. The spatial resolution of the raster layers used was 0.01° × 0.01° (approximately 1 km).

## 3 Results

### 3.1 Dominance and Ubiquity of Bacterial Genera Across Australia

**FIGURE 2:**
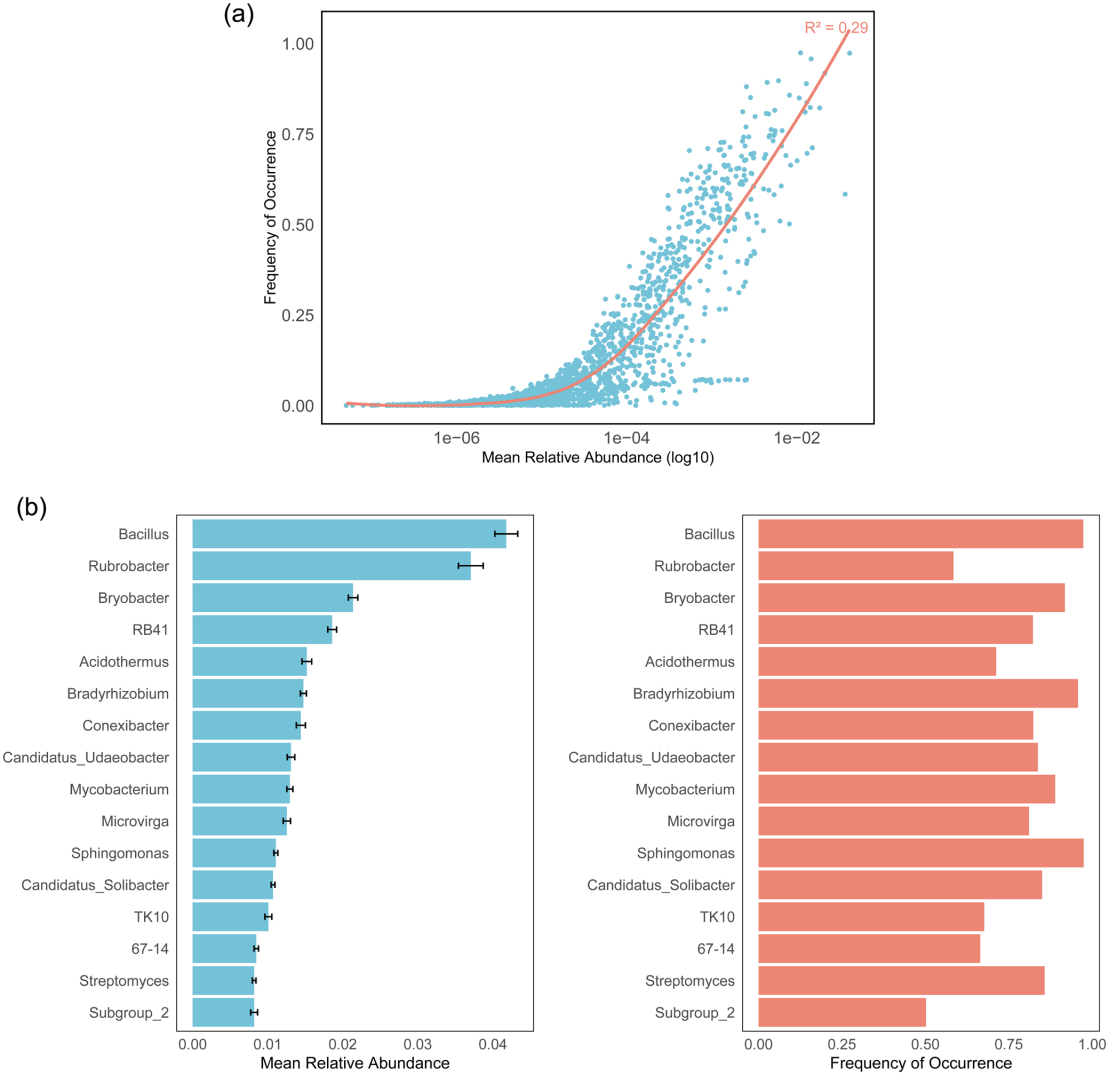
The mean relative abundance and frequency of the occurrence of the bacterial genus. (a) The relative abundance and frequency of total bacterial genus. (b) The relative abundance (left) and frequency (right) of dominant bacterial genera.

After processing the amplicon sequencing data and removing unclassified genera, we compiled a dataset of 1913 bacterial genera from the sampled sites to assess their abundance and occurrence (Figure 2). Of these, 159 genera were present in at least 50% of the sampling locations. Bacillus had the highest occurrence, detected in 97.4% of samples. In terms of mean relative abundance, 141 genera exceeded 0.1%, accounting for 55.2% of the total microbial community.

By considering both mean relative abundance and ubiquity, we identified the most dominant genera as those present in over 50% of samples with a mean relative abundance greater than 0.08%. This led to the selection of 16 dominant bacterial genera: Bacillus, Rubrobacter, Bryobacter, RB41, Acidothermus, Bradyrhizobium, Conexibacter, Candidatus Udaeobacter, Mycobacterium, Microvirga, Sphingomonas, Candidatus Solibacter, TK10, X67.14, Streptomyces, and Subgroup_2. Together, these genera accounted for 25.9% of the total relative abundance (Figure 2).

Among the dominant genera, Bacillus, Rubrobacter, and Bryobacter were the most prominent, with mean relative abundances of 4.18%, 3.71% and 2.14%, respectively. Notably, Rubrobacter was detected in only 58.4% of sampling points, suggesting a more specialised habitat preference. Sphingomonas, Bacillus and Bradyrhizobium were the most widespread, present in over 95% of sampled sites across Australia.

### 3.2 The Relationship Between the Pedo‐Climatic Factors and Bacterial Genus

**FIGURE 3:**
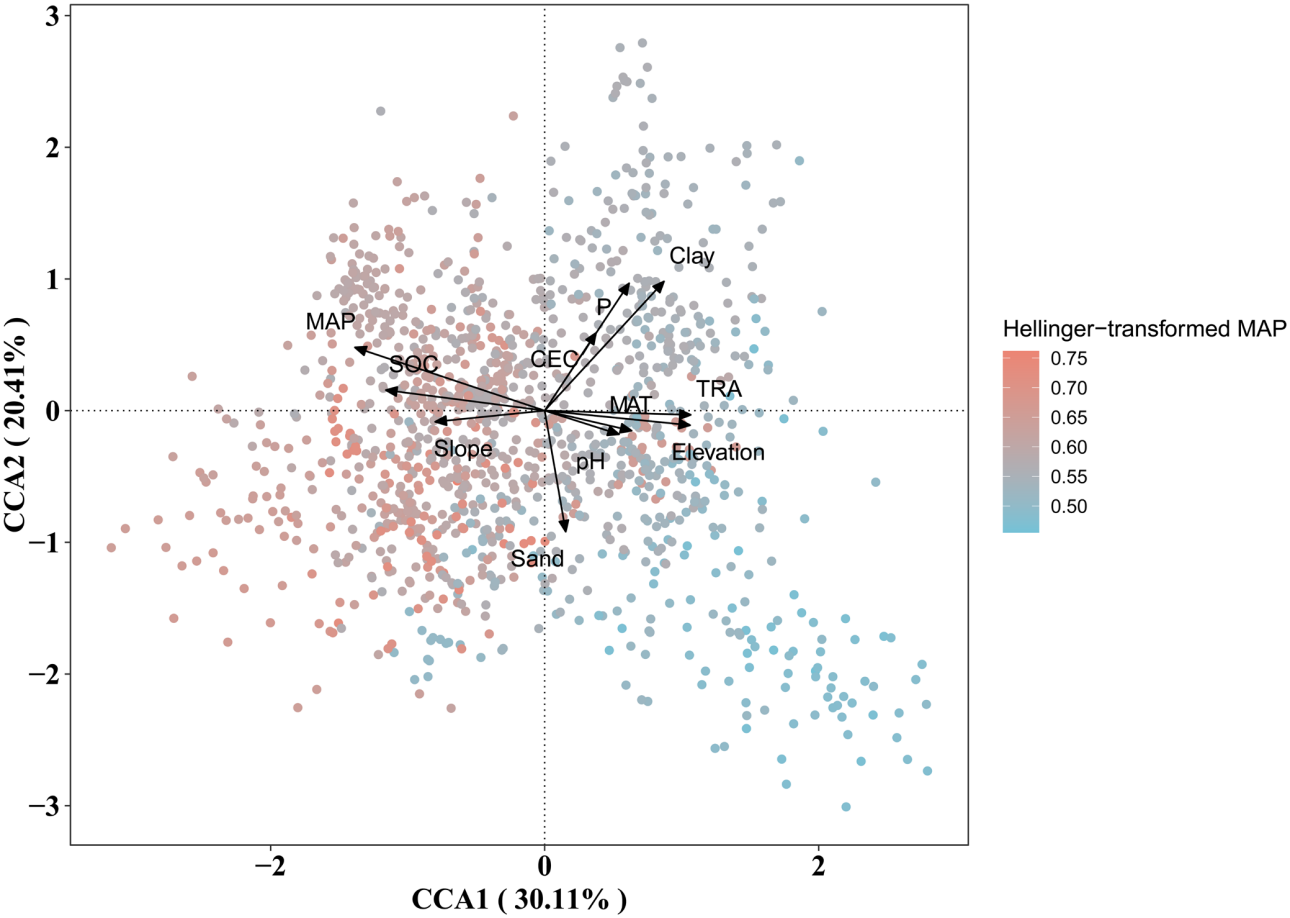
Ordination plot of the canonical correspondence analysis (CCA) to identify the relationships between bacterial genera communities and environmental factors. CEC, cation exchange capacity; MAT, annual daily mean temperature; MAP, mean annual precipitation; SOC, soil organic carbon; TRA, annual temperature range; P, total phosphorus.

To understand how pedo‐climatic factors influence bacterial genera, we conducted a CCA analysis. The first two axes explained 50.52% of the variance in bacterial community structure (Figure 3). Among the factors analysed, MAP emerged as the most significant determinant of bacterial genus composition (*r* ^2^ = 0.068, *p* < 0.001), followed by clay content (*r* ^2^ = 0.052, *p* < 0.001) and SOC (*r* ^2^ = 0.046, *p* < 0.001).

**FIGURE 4:**
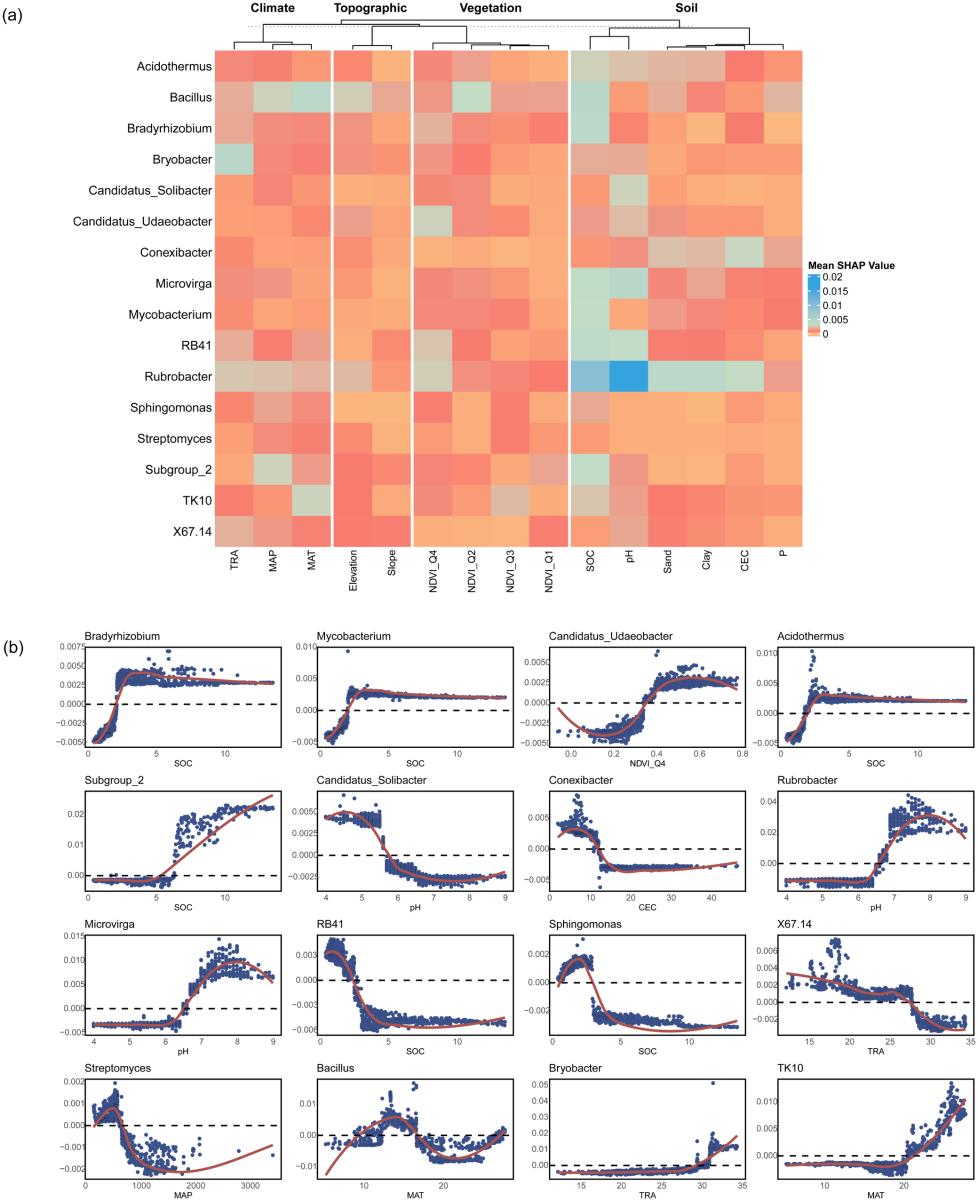
The results of the SHAP analysis. (a) Heatmap illustrating the SHAP values for multivariate variables across dominant bacterial genus communities. (b) Non‐linear patterns between pedo‐climatic factors and mean relative abundance (%) of dominant bacterial genera and their primary contributors chosen by the highest SHAP value. CEC, cation exchange capacity; EC, electric conductivity; MAP, mean annual precipitation; MAT, annual daily mean temperature; NDVI, Normalised Difference Vegetation Index; P, total phosphorus; SOC, soil organic carbon; TRA, annual temperature range.

We further explored the key determinants of dominant bacterial genera (Figure 4a). SHAP values were calculated to assess the impact of each variable on the predictions. The SHAP heatmap indicated that SOC and pH were the two most influential factors affecting the dominant bacterial genera (Figure 4b), followed by temperature and precipitation.

Higher SOC levels positively influenced the abundance of Acidothermus, Mycobacterium, Bradyrhizobium, and Subgroup_2. Acidothermus, Mycobacterium, and Bradyrhizobium were most prevalent in soils with SOC exceeding 2%, while Subgroup_2 thrived when SOC was above 5%. In contrast, RB41 and Sphingomonas preferred soils with SOC content below 3%. Soil pH also showed distinct effects on bacterial genera. Low pH conditions (below 5.5) favoured the survival of Candidatus Solibacter, whereas higher pH levels (above 6.5) promoted the growth of Microvirga and Rubrobacter.

### 3.3 Land Use Intensity Affects Soil Properties and the Dominant Bacterial Genera

**FIGURE 5:**
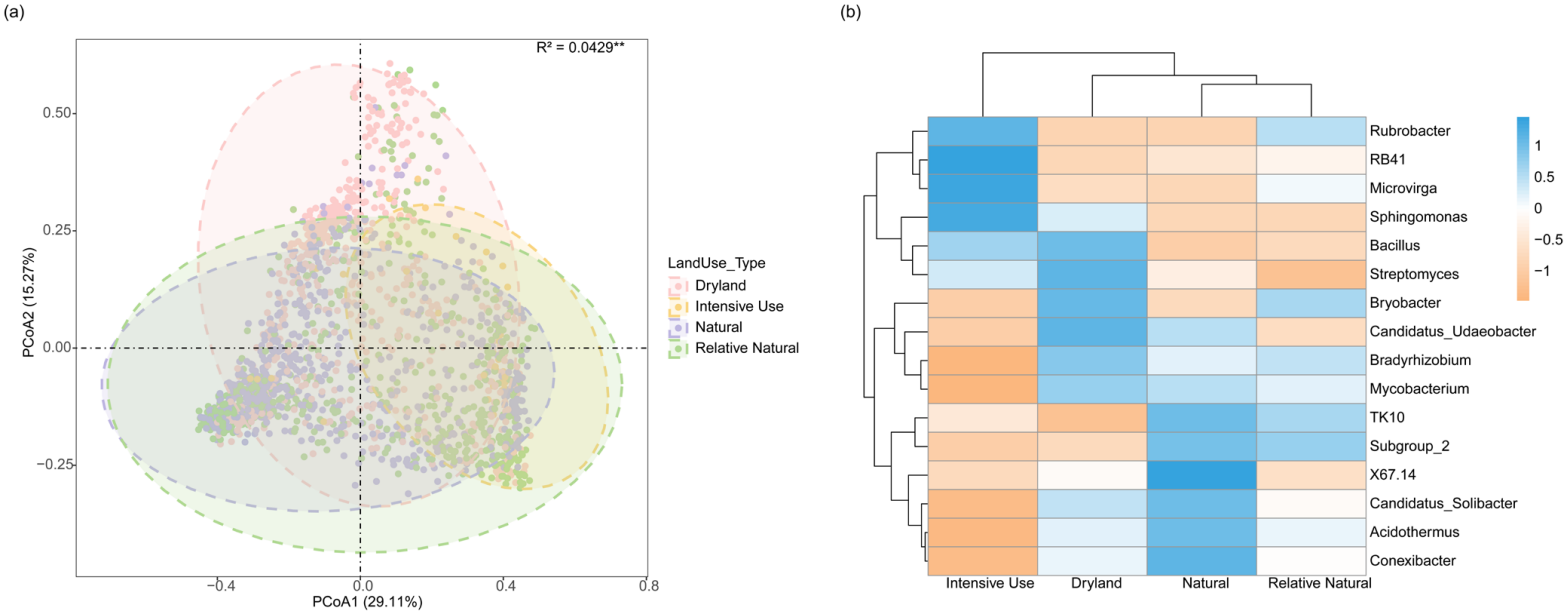
Land use effects on the dominant bacterial genera. (a) Principal coordinates analysis (PCoA) biplots based on the Bray–Curtis distance. (b) Heatmap of land use change affecting the dominant bacterial genera.

Land use significantly affected soil properties, with intensive land use causing more pronounced changes than other land‐use types (Figure S1). As land‐use intensity increased, SOC levels declined, while clay content, phosphorus, pH, and CEC tended to increase. Principal Coordinate Analysis revealed that land use significantly influenced the composition of dominant bacterial genera (*r* ^2^ = 0.04, *p* < 0.01; Figure 5a), with the first two axes accounting for 44.38% of the total variation. Heatmap clustering further highlighted distinct differences in bacterial community composition across land‐use types (Figure 5b). Bacterial communities in natural land‐use systems exhibited greater similarity and clustered closely with those in relatively natural environments, followed by dryland systems, and lastly, intensive land‐use areas.

Certain genera—such as TK10, Subgroup_2, X67.14, Candidatus Solibacter, Acidothermus, and Conexibacter—were more abundant in natural soils. In contrast, Rubrobacter, RB41, Microvirga, and Sphingomonas showed increased abundance in soils under intensive use.

### 3.4 Prediction of the Dominant Bacterial Distribution

**FIGURE 6:**
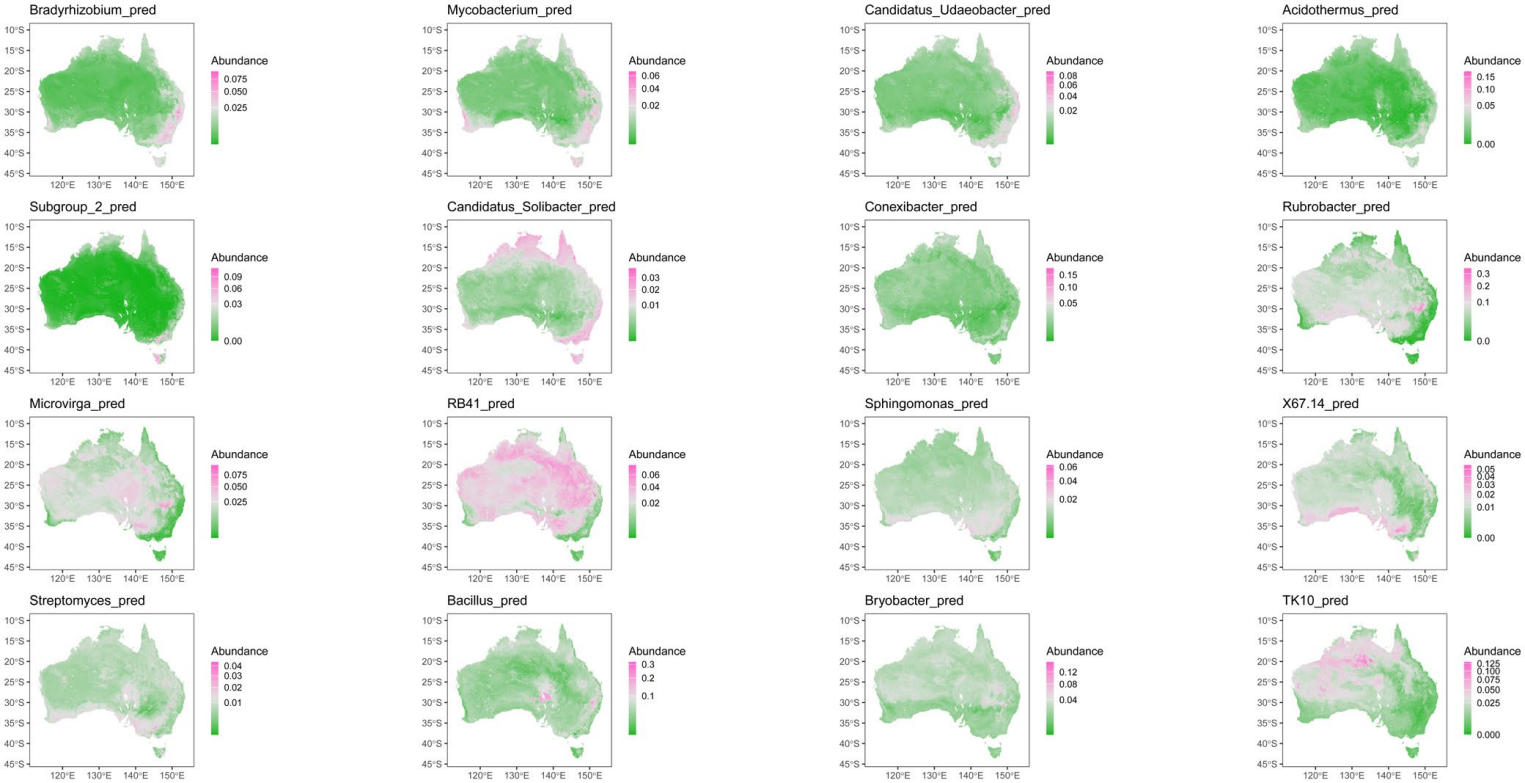
Predicted maps of the dominant soil bacteria genus distributions across Australia.

Based on the RF analysis results, we integrated spatial layers of soil properties, climatic variables, vegetation coverage, and topography to develop digital maps of dominant bacterial genera across Australia (Figure 6). The models demonstrated high accuracy, with most *R* ^2^ values exceeding 0.5 (Table S2).

The predicted maps revealed distinct spatial distribution patterns for each dominant bacterial genus. We performed an unsupervised classification using *k*‐means clustering to identify these patterns (Figure S2). Based on the clustering analysis, four unique spatial patterns emerged: coastal‐enriched, inland‐enriched, and two latitude‐related patterns corresponding to broad climatic zones.

In the coastal‐enriched group, genera such as Subgroup_2, Acidothermus, Bradyrhizobium, Mycobacterium, Candidatus Udaeobacter, and Candidatus Solibacter showed regional enrichments along the northern, southeastern, and southwestern coasts. For instance, Subgroup_2 had a predicted relative abundance of around 7% in the southeastern coastal region but less than 0.5% in central areas. The inland‐enriched group, including Microvirga, RB41, and Rubrobacter, was predominantly found in central Australia. Rubrobacter, in particular, had a predicted relative abundance exceeding 10% in most inland areas.

Other dominant bacterial genera displayed distinct latitude‐related distribution patterns. Sphingomonas, X67.14, and Streptomyces were more ubiquitous and abundant at higher latitudes, with hotspots along the southern coast but lower prevalence in southeastern Australia. Conversely, Bryobacter and TK10 showed higher relative abundances at lower latitudes, with Bryobacter concentrated in the east and TK10 more abundant in the northwest.

## 4 Discussion

We found the dominant bacterial genus in Australian soils, with four genera—Bacillus, Bryobacter, Bradyrhizobium, and Mycobacterium—ranking among the top 10 in both ubiquity and dominance. This finding aligns with previous research indicating that the most widespread bacterial species are also the most abundant (Karimi et al. 2018). Interestingly, although Rubrobacter ranked second in abundance, it was present in only 58.4% of samples, suggesting a preference for specific ecological niches. Our prediction maps further suggest that Rubrobacter may serve as an indicator of aridity, with its distribution predominantly concentrated in coastal regions (Chen et al. 2023). Besides, we observed significant differences when comparing bacterial genera distributions across countries, i.e., Australia and France. For example, comparing the top 10 genera from both countries, only Bacillus is shared, with its relative abundance slightly higher in Australia (4.2%) compared to France (3.8%) (Karimi et al. 2018). This discrepancy, partly due to methodological differences, likely underscores the influence of regional soil‐climatic and geographic factors on shaping bacterial communities (Du et al. 2025). These findings highlight the need for region‐specific studies to understand bacterial ecological preferences, which can support the development of global or continental microbial models.

Our research is the first to accurately identify distinct distribution patterns among dominant genera across Australia, including coastal versus inland enrichment and latitude‐related trends (Karimi et al. 2018). This trend not only occurs in the 16 dominant bacterial genera, but can also be found in other bacterial genera (Figure S3). Our results demonstrate that phylum‐level analyses may overlook important nuances in bacterial distribution. For example, within the Acidobacteria phylum, genera such as Bryobacter, RB41, Candidatus Solibacter, TK10, X67.14, and Subgroup_2 exhibited four distinct distribution patterns (Figure 5). This variation within a single phylum stresses the limitations of broad taxonomic approaches and emphasizes the need for investigating dominant genera independently to capture a more ecologically relevant picture of bacterial distribution (Xue et al. 2024). We also found that different environmental factors drive these distribution patterns. Coastal versus inland distributions are primarily shaped by soil pH and organic carbon (SOC), while latitude‐related patterns appear to be influenced by climatic factors (Figure 4b). Understanding these drivers can inform soil management strategies to address challenges posed by climate change.

Among the key environmental factors, pH was the major factor for the inland‐enriched pattern, while SOC dominated the coastal‐enriched pattern (Figure 4b). While the relationship between pH and bacterial diversity has been well documented (Rousk et al. 2010; Xue et al. 2024), our study brings new details by linking specific genera to particular pH niches. For example, Rubrobacter was most abundant in neutral to alkaline soils (pH 7–8), reflecting its optimal growth conditions (Norman et al. 2017). In contrast, Candidatus Solibacter and Acidothermus thrived in more acidic soils, consistent with their previously reported preferences (Oshkin et al. 2019; Rawat et al. 2012). The ability of these genera to adapt to such varied pH conditions may be linked to their cellular mechanisms for maintaining membrane stability, enzyme activity, and DNA integrity under challenging environmental conditions (Zhou et al. 2024). Additionally, our analysis showed that SOC content strongly influenced the distribution of Subgroup_2, Bradyrhizobium, Mycobacterium, and Acidothermus. These genera exhibited positive correlations with higher SOC levels, while RB41 showed a negative correlation, possibly due to different carbon utilization strategies (Fierer et al. 2007).

Regarding climate, our findings indicated that both temperature and precipitation drive the enrichment patterns at high latitudes, while temperature alone primarily influences enrichment at low latitudes. Higher temperatures were associated with greater abundances of Bryobacter and TK10, particularly in tropical regions, supporting previous findings that link microbial respiration rates to temperature (Carey et al. 2016). Precipitation also affected genera like Streptomyces, which declined in wetter environments, possibly due to water stress‐induced cell rupture (Schimel et al. 2007). These results highlight the interactions between climatic factors and microbial distribution, suggesting that temperature and precipitation drive latitude‐related patterns (Figure 4b). Understanding these relationships can help soil scientists predict bacterial responses to global climate change.

Increasing land use intensity significantly alters soil physicochemical properties, homogenising bacterial diversity and community composition (Figure 4), even at the higher taxonomic level (Figure S4). Natural and relatively natural regions exhibited the greatest bacterial similarity, whereas intensive land use resulted in the most significant divergence (Figure S5). This microbial homogenisation aligns with earlier reports (Gossner et al. 2016; Pino et al. 2023; Xue et al. 2024), where changes in soil properties—particularly declining SOC—led to shifts in bacterial communities. The reduction in Acidothermus, Mycobacterium, and Subgroup_2, along with increased abundance of RB41 in intensively used regions (Figure 5b), further supports this pattern. Changes in vegetation may also influence bacterial composition, as shifts in plant communities alter nutrient availability and rhizosphere interactions (Le Provost et al. 2021), with Candidatus Udaeobacter being notably sensitive to such changes (Figure 4b). These results indicate the need for sustainable land management practices to preserve microbial diversity and maintain soil health.

Understanding bacterial communities at a finer resolution enables soil scientists to implement more targeted soil management strategies in response to threats from climate change and human activities. While our study provides significant insights into the distribution of dominant bacterial genera and their environmental drivers, it is geographically limited to Australia. Future research should adopt a more targeted approach by specifically investigating key functional clades of bacteria, such as plant growth‐promoting bacteria or plant pathogens, as these groups have direct implications for land management strategies. Additionally, examining whether similar enrichment and distribution patterns apply to other regions and ecosystems, and integrating other soil organisms, such as fungi and protists, could provide a more holistic understanding of microbial communities and their roles in soil ecosystems.

## 5 Conclusion

This study addressed the gap in understanding soil bacterial distribution at the genus level on a continental scale. We developed predictive models integrating soil and climatic factors, providing robust evidence for bacterial distribution across Australia. Our models highlighted that pedo‐climatic factors—including soil properties (particularly pH and SOC), climatic conditions, vegetation indices, and topographic variables—significantly explain and predict the distribution of dominant bacterial genera. The predicted hotspot maps revealed four important distribution patterns: coastal‐enriched, inland‐enriched, low‐latitude‐enriched, and high‐latitude‐enriched. These biogeographic patterns and their environmental controls offer valuable insights into soil microbial ecology, providing essential information on microbial mechanisms at large scales. Such detailed mapping enhances our ability to predict shifts in bacterial communities in response to environmental changes, land use, and climate variability, providing a robust framework for soil health monitoring and conservation efforts. The models could be used to help land managers identify bacterial hotspots, enabling them to develop tailored soil management strategies that maintain or enhance microbial diversity and ecosystem services, such as nutrient cycling and carbon sequestration. The identified distribution patterns could also guide targeted efforts for soil restoration and biodiversity conservation, particularly in regions at risk of degradation.

## Author Contributions

Mingming Du designed the study, conducted molecular work, performed statistical analyses, and led the writing and editing. Peipei Xue contributed to molecular work, statistical analyses, and manuscript editing. Budiman Minasny and Alex McBratney contributed to study design and manuscript editing. Vanessa Pino, Patrice de Caritat contributed to molecular work and manuscript editing. Mario Fajardo Pedraza, Ji‐Zheng He, Qinglin Chen and Andrew Bissett contributed to molecular work.

## Conflicts of Interest

The authors declare no conflicts of interest.

## Supporting information


Data S1:

## Data Availability

The data that support the findings of this study are available in the BioPlatforms Australia project's data portal at http://doi.org/10.4227/71/561c9bc670099, the Geoscience Australia repository at https://doi.org/10.11636/Record.2011.020, the National Centre for Biotechnology Information (NCBI) https://www.ncbi.nlm.nih.gov/bioproject/PRJNA659980/, https://www.ncbi.nlm.nih.gov/bioproject/PRJNA729592/, and https://www.ncbi.nlm.nih.gov/sra/PRJNA1281203. Additional code is available on the github https://github.com/Mingming‐Du/Australia_Genus.git.
