# Supplementary material for: Soil Microbial Blueprint: Predicting Soil Dominant Bacterial Genera Distribution Across Australia
Source: Mol Ecol. 2025 Oct 4;34(21):e70135. doi: 10.1111/mec.70135 (PMC12573719; doi:10.1111/mec.70135)
Supplement: Supplementary file 1 — Data S1: [file MEC-34-e70135-s001.docx]

## Supplementary Material

Figures:


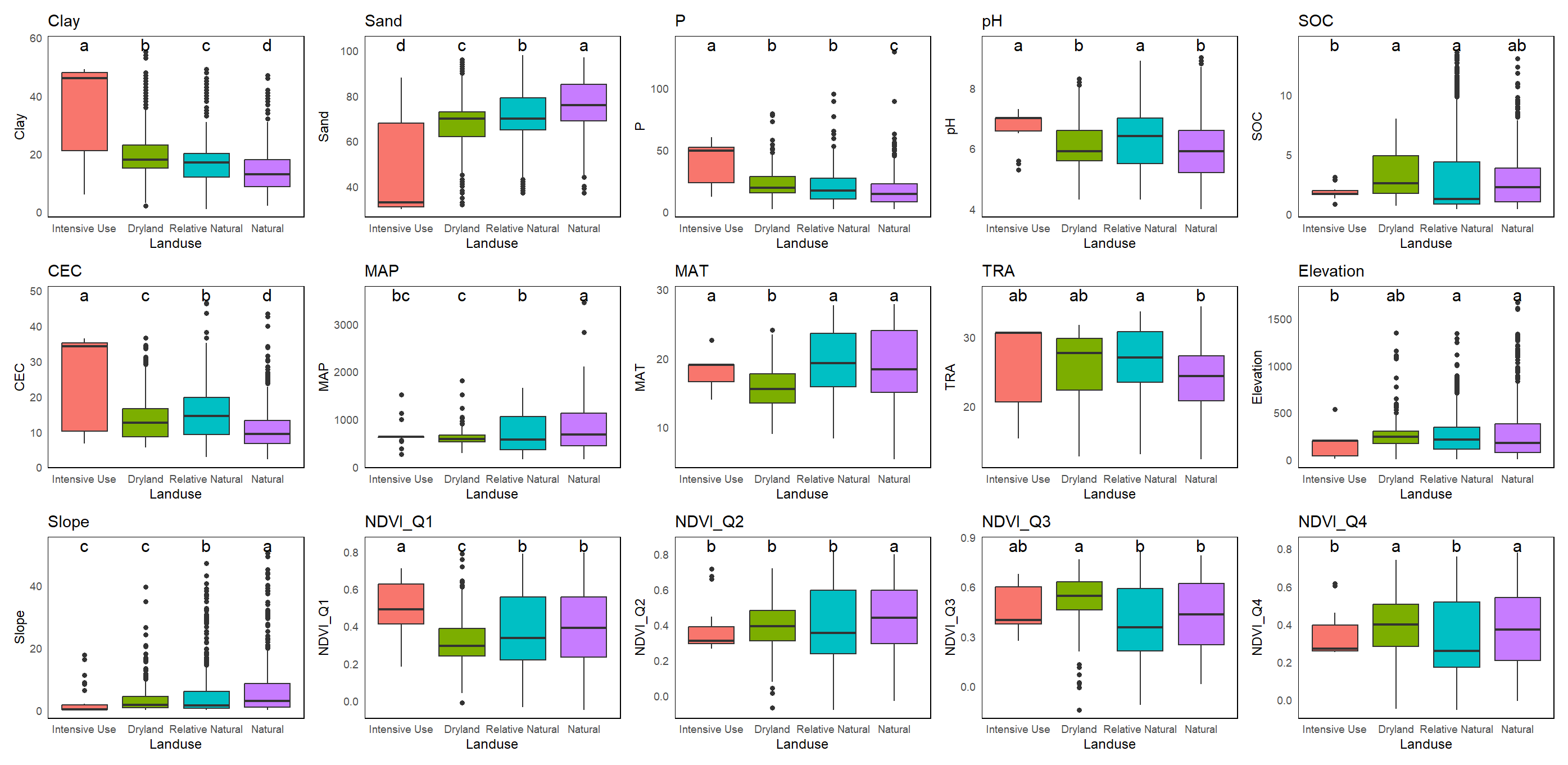


Fig. S1. Boxplots of different pedo-climatic properties as under different land uses. Statistical comparisons were performed by one-way ANOVA followed by Tukey’s honest significant difference (HSD) test. Groups sharing the same lowercase letter are not significantly different; different letters indicate pairwise differences (*p* < 0.05). CEC, cation exchange capacity; MAT, annual daily mean temperature; MAP, annual precipitation; TRA, annual temperature range; EC, electric conductivity; SOC, soil organic carbon; P, total phosphorus; NDVI, Normalized Difference Vegetation Index.


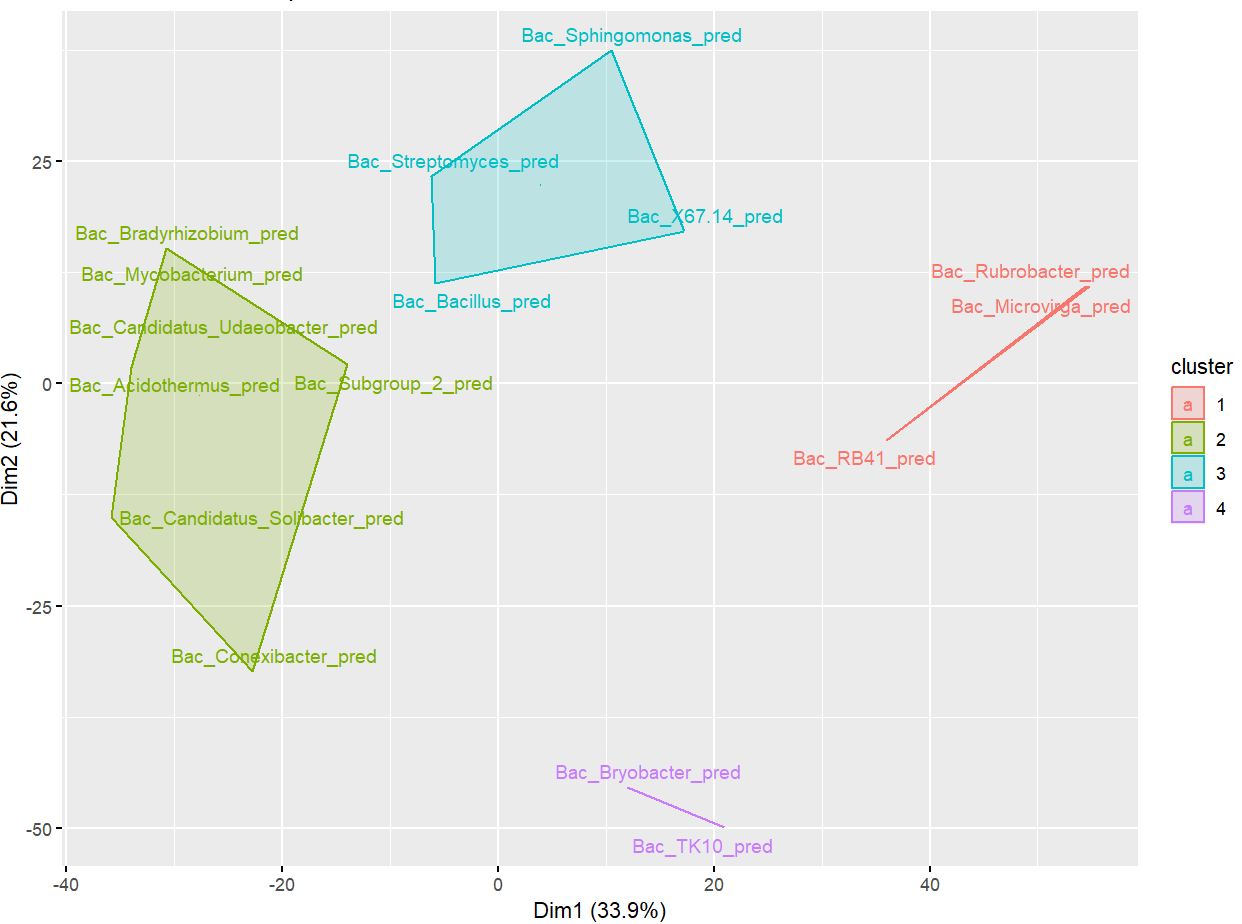


Fig. S2. K-means clustering of the dominant bacterial genera distribution patterns. Each point represents one dominant genus. Colored by its cluster membership: (1) Inland-enriched genera, (2) Coastal-enriched genera, (3) High-latitude enriched genera, (4) Low-latitude enriched genera


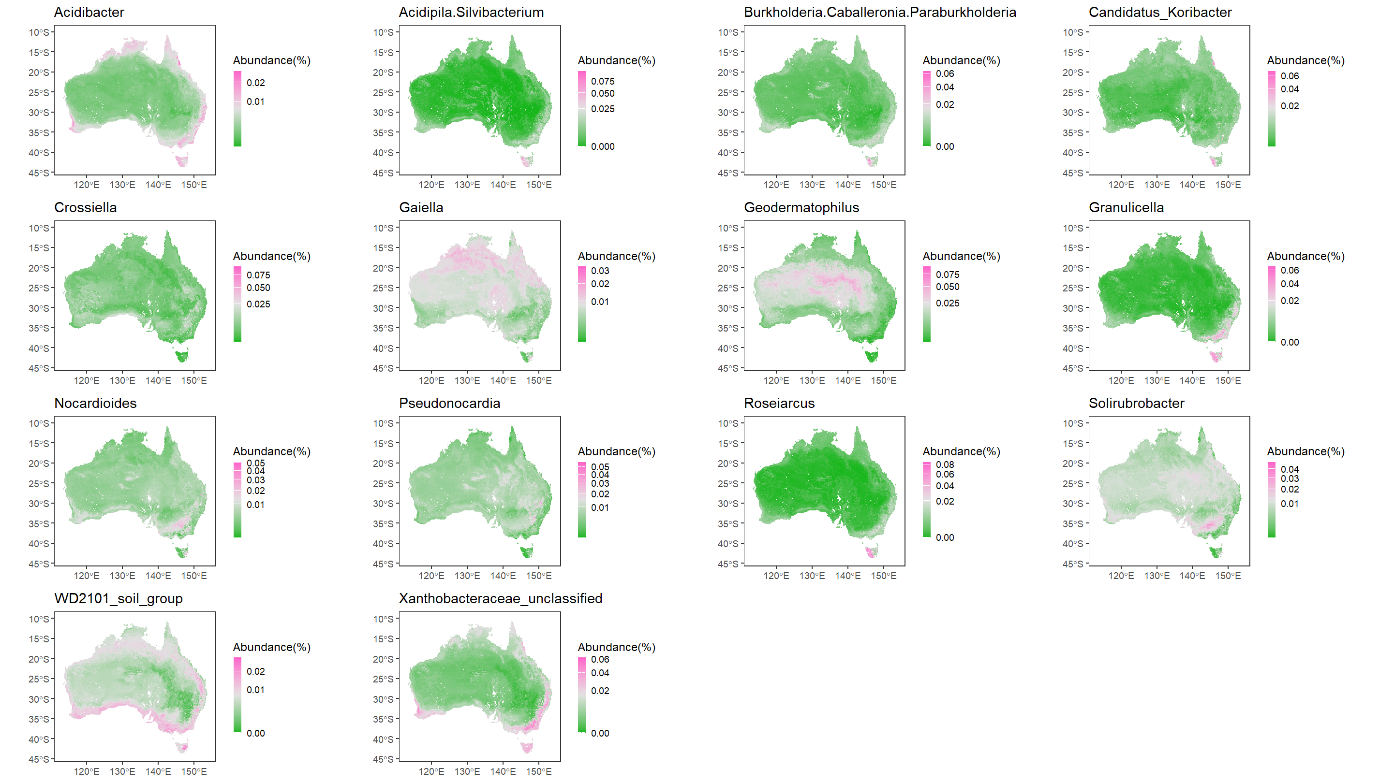


Fig. S3. Predicted maps of the soil bacteria genus distributions with relative abundance higher than 0.5% across Australia.


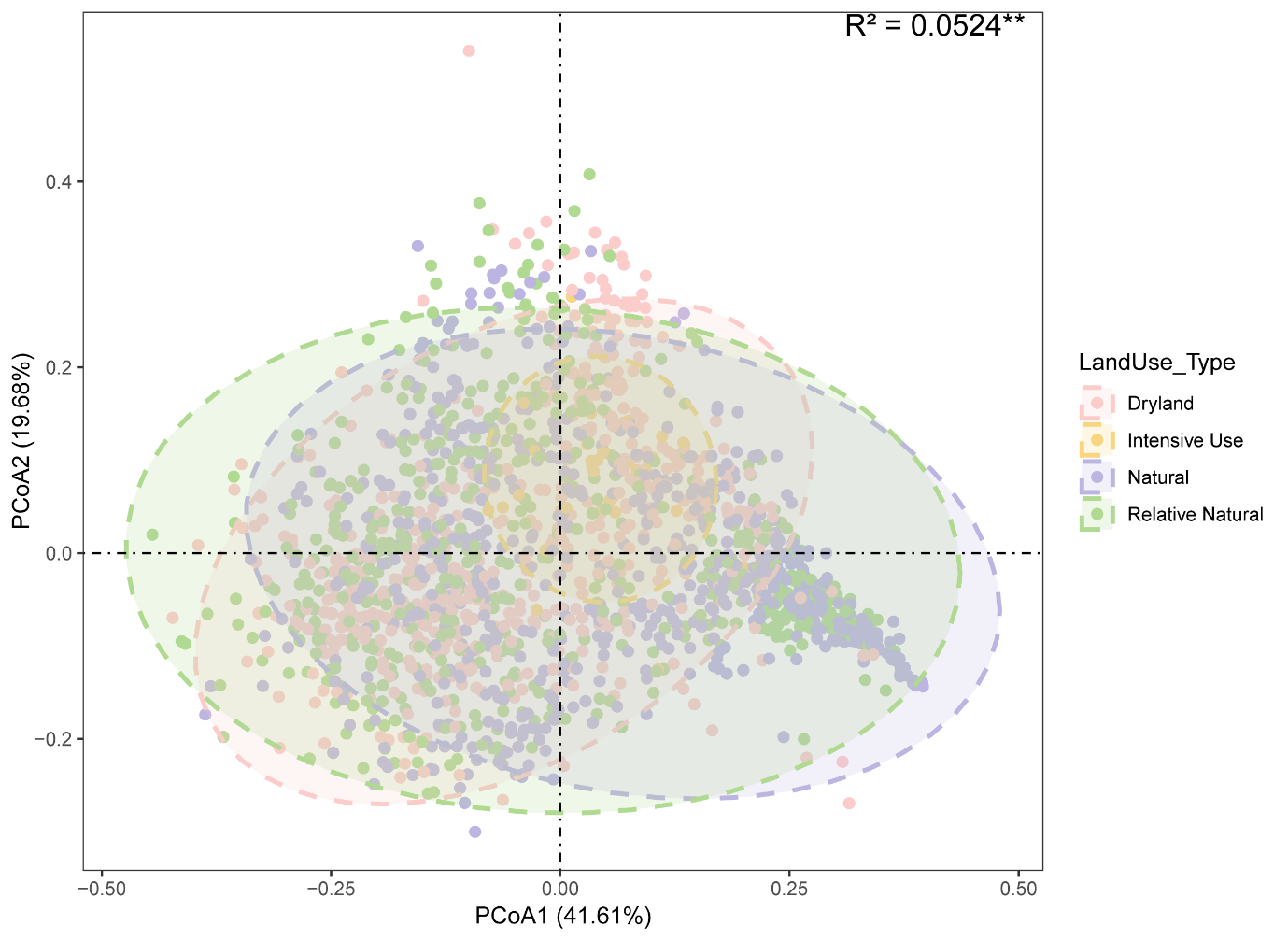


Fig. S4. Land use effects on the bacterial phylum-level communities. Principal Coordinates Analysis (PCoA) biplots based on the Bray-Curtis distance.


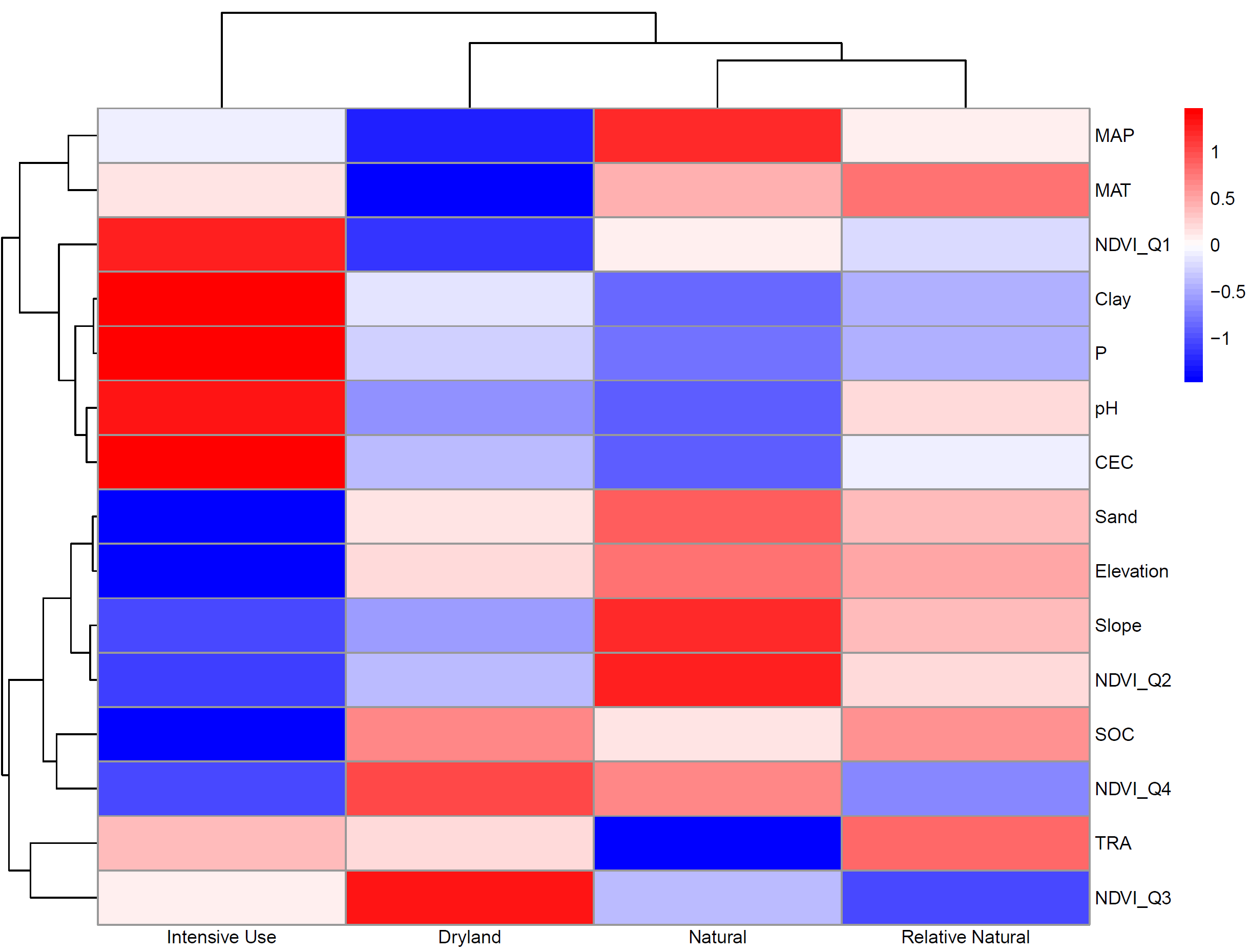


Fig. S5. Heatmap of land use type effects on soil properties and climatic factors. CEC, cation exchange capacity; MAT, annual daily mean temperature; MAP, annual precipitation; TRA, annual temperature range; EC, electric conductivity; SOC, soil organic carbon; P, total phosphorus; NDVI, Normalized Difference Vegetation Index.

## Tables

Table S1. Land use classification

| Land Use Classification | Categories |
| --- | --- |
| Natural | "Strict nature reserves," "National park," "Habitat/species management area," "Wilderness area," "Natural feature protection," "Protected landscape," "Other conserved area," "Residual native cover," "Other minimal use," "Managed resource protection," "Traditional indigenous uses," and "Defence land - natural areas." |
| Relative natural | "Grazing native vegetation" and "Production forestry." |
| Dryland | "Grazing modified pastures," "Cereals," "Pulses," "Cotton," "Plantation forestry," "Grapes," "Hay and silage," "Oil seeds," and "Sugar." |
| Intensive use | "Irrigated cotton," "Irrigated grapes," "Irrigated tree fruits," and "Grazing irrigated modified pastures." |

Table S2. Validation statistics of the quantile regression forest models for the dominant bacterial genus obtained by random 10-fold cross-validation. RMSE: Root Mean Squared Error, R^2^: coefficient of determination, and rhoC: Concordance Correlation Coefficient.

| Genus | Five databases | | |
| --- | --- | --- | --- |
|  | RMSE | R^2^ | rhoC |
| Bacillus | 0.05 | 0.39 | 0.59 |
| Rubrobacter | 0.04 | 0.77 | 0.87 |
| Bryobacter | 0.02 | 0.70 | 0.82 |
| RB41 | 0.02 | 0.48 | 0.67 |
| Acidothermus | 0.02 | 0.54 | 0.7 |
| Bradyrhizobium | 0.01 | 0.75 | 0.86 |
| Conexibacter | 0.02 | 0.56 | 0.71 |
| Candidatus_Udaeobacter | 0.02 | 0.45 | 0.64 |
| Mycobacterium | 0.01 | 0.58 | 0.73 |
| Microvirga | 0.01 | 0.64 | 0.78 |
| Sphingomonas | 0.01 | 0.48 | 0.66 |
| Candidatus_Solibacter | 0.01 | 0.52 | 0.69 |
| TK10 | 0.01 | 0.64 | 0.76 |
| X67.14 | 0.01 | 0.66 | 0.78 |
| Streptomyces | 0.01 | 0.31 | 0.51 |
| Subgroup_2 | 0.01 | 0.75 | 0.85 |
